# Second Cancer Incidence and Cause‐Specific Mortality in Primary Gastrointestinal Non‐Hodgkin Lymphoma Survivors: A Population‐Based Cohort Study

**DOI:** 10.1002/cam4.71405

**Published:** 2025-11-20

**Authors:** Jiahe Wu, Jiangping Yang, Yuxi Ding, Jili Wang, Xiaofei Cheng, Liangshun You, Feng Zhao

**Affiliations:** ^1^ Department of Radiation Oncology, The First Affiliated Hospital Zhejiang University School of Medicine Hangzhou Zhejiang People's Republic of China; ^2^ Graduate School Zhejiang University School of Medicine Hangzhou Zhejiang People's Republic of China; ^3^ Cancer Institute (Key Laboratory of Cancer Prevention and Intervention, China National Ministry of Education) of the Second Affiliated Hospital and Institute of Translational Medicine Zhejiang University School of Medicine Hangzhou Zhejiang People's Republic of China; ^4^ Cancer Center of Zhejiang University Hangzhou Zhejiang People's Republic of China; ^5^ School of Basic Medical Sciences and Forensic Medicine, Hangzhou Medical College Hangzhou Zhejiang People's Republic of China; ^6^ Department of Pathology, The First Affiliated Hospital Zhejiang University School of Medicine Hangzhou Zhejiang People's Republic of China; ^7^ Department of Colorectal Surgery, the First Affiliated Hospital Zhejiang University School of Medicine Hangzhou Zhejiang People's Republic of China; ^8^ Department of Hematology, The First Affiliated Hospital Zhejiang University School of Medicine Hangzhou Zhejiang People's Republic of China

**Keywords:** cause‐specific mortality, primary gastrointestinal non‐Hodgkin lymphoma, second primary cancers, standardized incidence ratio, standardized mortality ratio

## Abstract

**Background:**

Therapeutic advances in primary gastrointestinal non‐Hodgkin lymphoma (PGI‐NHL) have improved patient prognosis but also raised concerns regarding long‐term health outcomes. This study assesses patterns of second primary cancers (SPCs) and cause‐specific mortality in PGI‐NHL survivors.

**Methods:**

A total of 7556 six‐month survivors of PGI‐NHL diagnosed between 1975 and 2015 were identified from the SEER database. SPC risks were evaluated using overall and site‐specific standardized incidence ratios (SIRs) and absolute excess risks (AERs) relative to the US general population. Cause‐specific mortality was assessed through proportional death distribution, the Fine–Gray competing risk regression model, standardized mortality ratios (SMRs), and AERs.

**Results:**

During 75,914 person‐years of follow‐up, 1245 patients developed 1412 SPCs (SIR = 1.20, 95% CI = 1.14–1.27). Significantly elevated SIRs were observed for solid tumors (SIR = 1.16, 95% CI = 1.10–1.23) and hematolymphoid malignancies (SIR = 1.82, 95% CI = 1.49–2.19). Survivors with over 20 years of follow‐up faced increased risks of oral cavity and pharyngeal cancers (SIR = 2.76, 95% CI = 1.11–5.69), gastric cancer (SIR = 3.12, 95% CI = 1.15–6.80), pancreatic cancer (SIR = 2.40, 95% CI = 1.10–4.55), and leukemia (SIR = 2.57, 95% CI = 1.17–4.87). The risk of developing SPCs decreased with increasing age at PGI‐NHL diagnosis. Patients receiving combined chemoradiotherapy exhibited the highest overall SPC risk (SIR = 1.32, 95% CI = 1.09–1.58), particularly for gastric cancer (SIR = 8.20, 95% CI = 4.59–13.53) and pancreatic cancer (SIR = 3.15, 95% CI = 1.44–5.98). A total of 4949 deaths occurred (SMR = 1.80, 95% CI = 1.75–1.85), with excess mortality persisting beyond two decades. The cumulative incidence of all‐cause mortality was 28.9% at 5 years, 45.8% at 10 years, and 69.1% at 20 years post‐diagnosis. Non‐cancer causes posed a significant risk, with higher mortality from infections (SMR = 2.48, 95% CI = 2.22–2.76), chronic liver diseases (SMR = 1.72, 95% CI = 1.20–2.38), and benign/in situ neoplasms (SMR = 1.62, 95% CI = 1.05–2.38).

**Conclusions:**

PGI‐NHL survivors face elevated long‐term SPC risk, particularly after chemoradiotherapy and in younger patients. Excess risks for both SPCs and mortality persist beyond two decades, necessitating lifelong, risk‐adapted survivorship care that includes cancer surveillance, infection prevention, and chronic comorbidity management.

## 1 Introduction

Primary extranodal non‐Hodgkin's lymphoma (PEN‐NHL) is a heterogeneous group of malignancies with distinct natural histories, therapeutic responses, and prognoses compared to nodal NHL [1]. Among PEN‐NHL categories, primary gastrointestinal non‐Hodgkin's lymphoma (PGI‐NHL) is the most common, representing 30%–40% of PEN‐NHL cases but only 1%–4% of all gastrointestinal malignancies [2, 3, 4]. Most PGI‐NHLs arise in the stomach and small intestine, exhibiting a clinical spectrum from indolent localized lesions to aggressive metastatic disease [4, 5]. Histologically, 70%–80% of PGI‐NHL cases are classified as diffuse large B‐cell lymphoma (DLBCL) or extranodal marginal zone lymphoma of mucosa‐associated lymphoid tissue (MALT) [4, 6]; advancements in diagnostic techniques have also enabled the detection of rarer subtypes, such as diminutive follicular lymphoma [7, 8].

Therapeutic innovations, including the introduction of *Helicobacter pylori* eradication therapy in the 1990s and the widespread use of rituximab since the early 2000s, have significantly improved survival in PGI‐NHL patients [9, 10, 11]. However, prolonged survivorship heightens concerns about second primary cancers (SPCs), which may arise from shared etiologies, host susceptibility, or treatment‐related carcinogenesis, potentially offsetting survival gains [12, 13]. Although increased SPC risk has been reported in broader NHL populations, focusing on PGI‐NHL is crucial due to its unique carcinogenic pathways—driven by chronic inflammation and infections like *H. pylori* [14, 15]. This specific environmental context, combined with the characteristic treatment modalities for PGI‐NHL—often involving surgery, abdominal radiotherapy, chemotherapy, and immunotherapy—may distinctly influence its profile of late effects [2]. Despite this, research on SPC risk in PGI‐NHL survivors is limited, largely confined to small cohorts with restricted follow‐up periods [16, 17, 18, 19]. Additionally, with extended survival, non‐cancer mortality attributed to cumulative treatment toxicity and the progression of comorbidities has become an increasingly important cause of death. To our knowledge, no study has systematically assessed cause‐specific mortality in PGI‐NHL patients. A comprehensive analysis of mortality patterns is urgently needed to optimize surveillance and guide survivorship care.

Therefore, we conducted a population‐based analysis of 7556 six‐month survivors of PGI‐NHL using the US SEER cancer registry database (1975–2015). This study systematically evaluated the incidence of SPCs and cause‐specific mortality, stratified by patient characteristics, treatment modalities, and latency periods. Our findings provide evidence on the underlying factors that may influence future cancer risk profiles and survival outcomes, complementing the sparse existing literature and laying the foundation for tailored management strategies.

## 2 Methods

### 2.1 Data Source and Study Population

The data for this study were obtained from the National Cancer Institute's SEER 8 Registries Database, which captures cancer incidence and survival data from specific geographic areas, covering approximately 8.3% of the US population. We identified adults (≥ 18 years) diagnosed with PGI‐NHL as the first primary cancer between January 1, 1975, and December 31, 2015 using ICD‐O‐3 diagnosis codes for extranodal NHL and primary site codes for stomach (C16.0–C16.9), small intestine (C17.0–C17.9), colon (C18.0–C18.9), and rectum (C19.9, C20.9). We observed PGI‐NHL survivors for subsequent primary malignancies starting 6 months after the PGI‐NHL diagnosis (6‐month latency exclusion period) to prevent the inclusion of synchronous tumors or metastases. Patients who died or were lost to follow‐up prior to this period were excluded from all person‐years‐based analyses. Additionally, individuals with unknown survival status or those diagnosed solely through autopsy or death certificate were excluded from the study. Non‐Hodgkin lymphoma and nonmelanoma skin cancers occurring as second malignancies were also excluded. Follow‐up continued until the occurrence of SPC incidence, death, loss to follow‐up, or the study end date (December 31, 2021), whichever came first.

### 2.2 Statistical Analyses

The SEER*Stat Multiple Primary‐SIR tool (version 8.4.5) was used to calculate the standardized incidence ratio (SIR) and absolute excess risk (AER). SIRs were defined as the ratio of observed to expected SPCs, estimating the relative risk of SPCs among PGI‐NHL survivors compared to the general US population. Expected SPC counts were derived by multiplying gender‐, race‐, age‐, and calendar year‐specific incidence rates from the 2000 US standard population by the cohort's accrued person‐years at risk. AERs quantified the absolute excess burden of SPCs among cancer survivors, expressed as extra events per 10,000 person‐years compared to the matched general population.

The risk of developing SPCs was assessed in the entire PGI‐NHL survivor cohort, as well as stratified by sex (female or male), race (White, Black, or other/unknown), calendar year of diagnosis (1975–1984, 1985–1994, 1995–2004, 2005–2015), age group at diagnosis (18–49, 50–74, ≥ 75 years), Ann Arbor stage (I–IV, unknown), primary anatomic site (stomach, small intestine, colon, rectum), histologic subtype (DLBCL, MALT lymphoma, follicular lymphoma, or other specified/unspecified), latency period (0.5–4 years, 5–9 years, 10–19 years, ≥ 20 years), and initial treatment modality. Treatment was classified into four mutually exclusive groups: radiotherapy (RT) without chemotherapy (CT), CT without RT, chemoradiotherapy (CRT), and surgery alone (no RT/CT). Statistical tests and 95% confidence intervals (CIs) for overall and site‐specific relative risks of SPCs were based on the assumption that the observed number of second cancers followed a Poisson distribution. Tests for heterogeneity and linear trends were performed using the methods of Breslow et al. [20] Additionally, the Fine and Gray model was used to calculate the cumulative incidence of SPCs at 5, 10, and 20 years of follow‐up, accounting for the competing risk of death.

We estimated the relative risk of death using the standardized mortality ratio (SMR), calculated as the ratio of observed deaths in the cohort to expected deaths based on gender‐, race‐, age‐, and calendar year‐specific US general population mortality rates. We also calculated the proportion of deaths and cumulative mortality for comparisons across different causes of death. Deaths were categorized into three major classes: NHL‐related deaths, SPC‐related deaths, and deaths from non‐cancer causes. The proportion of deaths was defined as cause‐specific mortality relative to overall mortality to identify the most prevalent cause. The cumulative risk of different causes of death and their competing relationships were quantified by plotting cumulative incidence curves using competing‐risk regression based on the Fine–Gray model. All statistical analyses were performed using R software (version 4.4.1), with a two‐sided *p*‐value < 0.05 considered statistically significant.

## 3 Results

A total of 7556 patients with PGI‐NHL met the eligibility criteria and were included in the study. This cohort, which comprised 3438 10‐year survivors and 1026 20‐year survivors, collectively contributed 75,914 person‐years of follow‐up. Selected baseline and treatment characteristics of the cohort are summarized in Table 1. The median age at diagnosis of PGI‐NHL was 65 years. During follow‐up, 1412 SPCs were diagnosed in 1245 patients. The cumulative incidence of any SPC at 5, 10, and 20 years following the diagnosis of PGI‐NHL was 6.0% (95% CI = 5.5%–6.6%), 11.4% (95% CI = 10.7%–12.1%), and 17.8% (95% CI = 16.8%–18.7%), respectively.

**TABLE 1:** Characteristics of 7556 six‐month survivors of PGI‐NHL reported to the SEER Program (1975–2015).

Patient characteristics	No. of patients	Person‐years of follow‐up	No. of second cancers	SIR (95% CI)	AER
**Total**	7556	75,914	1412	1.20 (1.14, 1.27)*	31.07
**Sex**					
Males	4241	40,979	872	1.22 (1.14, 1.30)*	37.93
Females	3315	34,935	540	1.18 (1.08, 1.28)*	23.01
**Year of PGI‐NHL diagnosis**					
1975–1984	850	9705	150	0.98 (0.83, 1.15)	−2.80
1985–1994	1616	18,014	348	1.18 (1.06, 1.31)*	29.44
1995–2004	2256	25,535	484	1.22 (1.12, 1.34)*	34.53
2005–2015	2834	22,661	430	1.29 (1.17, 1.42)*	42.95
**Age at PGI‐NHL diagnosis, years**					
18–49	1408	20,085	178	1.46 (1.26, 1.70)*	28.06
50–74	4190	44,412	952	1.21 (1.14, 1.29)*	37.26
≥ 75	1958	11,418	282	1.05 (0.93, 1.18)	12.25
**Race**					
White	6074	60,778	1198	1.20 (1.13, 1.27)*	32.79
Black	446	4589	65	1.05 (0.81, 1.34)	7.29
Other/unknown	1036	10,547	149	1.29 (1.09, 1.51)*	31.45
**Stage**					
I	3669	38,195	698	1.15 (1.07, 1.24)*	24.16
II	1530	15,702	280	1.19 (1.05, 1.33)*	27.82
III	270	2169	42	1.25 (0.90, 1.69)	38.52
IV	1084	9102	204	1.54 (1.33, 1.76)*	78.39
Unknown	1003	10,746	188	1.12 (0.97, 1.29)	18.76
**Site of PGI‐NHL**					
Stomach	4134	41,246	818	1.23 (1.15, 1.32)*	37.05
Small intestine	1955	20,234	361	1.20 (1.08, 1.33)*	29.42
Colon	1161	11,635	183	1.07 (0.92, 1.24)	10.46
Rectum	306	2799	50	1.29 (0.96, 1.70)	40.42
**PGI‐NHL histopathology**					
DLBCL	3364	32,290	626	1.24 (1.15, 1.34)*	37.67
MALT lymphoma	1635	16,794	311	1.20 (1.07, 1.34)*	30.75
Follicular and nodular NHL	738	8669	159	1.14 (0.97, 1.33)	22.60
Others, specified	835	7906	140	1.21 (1.02, 1.42)*	30.35
Unspecified cell type	984	10,255	176	1.12 (0.96, 1.30)	18.50
**Follow‐up interval, years**					
0.5–4	7556	27,511	466	1.16 (1.05, 1.27)*	22.74
5–9	5357	22,040	432	1.27 (1.15, 1.39)*	41.50
10–19	3438	20,364	393	1.19 (1.08, 1.31)*	30.70
≥ 20	1026	5999	121	1.19 (0.99, 1.42)	32.16
**Treatment exposures**					
Untreated/treatment status unknown	1304	12,333	227	1.15 (1.00, 1.30)	23.35
Only surgery	1377	15,176	255	0.99 (0.87, 1.12)	−2.36
RT, no CT	780	8252	159	1.27 (1.08, 1.48)*	40.97
CT, no RT	3497	34,062	655	1.29 (1.20, 1.40)*	43.64
CRT	598	6091	116	1.32 (1.09, 1.58)*	46.22

Abbreviations: AER, absolute excess risk; CRT, Chemoradiotherapy; CT, chemotherapy; DLBCL, diffuse large B‐cell lymphoma; MALT, mucosa‐associated lymphoid tissue; PGI‐NHL, primary gastrointestinal non‐Hodgkin lymphoma; RT, radiotherapy; SEER, Surveillance, Epidemiology, and End Results; SIR, standardized incidence ratio.

* Statistically significant standardized incidence ratios are indicated.

The risk of SPCs among PGI‐NHL patients was significantly higher than that in the US general population (SIR = 1.20, 95% CI = 1.14–1.27), resulting in 31.07 excess cancers per 10,000 person‐years. The overall risk of SPCs was comparable between male and female patients (SIR = 1.22 [95% CI = 1.14–1.30] versus 1.18 [95% CI = 1.08–1.28], *p*_heterogeneity_ = 0.45), whereas a significantly elevated risk of hematolymphoid malignancies occurred exclusively in men (SIR = 2.09, 95% CI = 1.66–2.61). An upward trend in SPC incidence was observed among PGI‐NHL patients diagnosed in more recent years (*p*_trend_ = 0.006). Analysis by treatment era (pre‐2001 vs. post‐2001) further revealed a significant increase in overall SPC risk in the modern cohort (SIR = 1.29, 95% CI = 1.19–1.39) compared to the earlier cohort (SIR = 1.14, 95% CI = 1.06–1.22; *p*_heterogeneity_ = 0.021; Figure S1). Notably, the SIR for thyroid cancer increased markedly from 0.73 (95% CI = 0.20–1.88) to 2.29 (95% CI = 1.28–3.78) (*p*_heterogeneity_ = 0.026). Similarly, the SIR for leukemia rose significantly from 1.51 (95% CI = 1.02–2.14) to 2.70 (95% CI = 1.98–3.60) (*p*_heterogeneity_ = 0.011). In the analysis stratified by anatomic site or histologic subtype of PGI‐NHL, although significantly increased SIRs for SPCs were confined to specific sites or subtypes (as shown in Table 1), no statistical difference in overall SPC risk was observed among subgroups within either of these two strata (*p*_heterogeneity_ = 0.36 for anatomic sites; *p*_heterogeneity_ = 0.75 for histologic subtypes). A detailed analysis of SPC risks across the major histologic subtypes is presented in Figure S2, which delineates the specific risk profiles for each subtype. For the various SPC sites analyzed, slight differences in SPC risk were observed among histologic subtypes; however, none of these site‐specific variations reached statistical significance. Additionally, evaluation of SPC risk by PGI‐NHL stage at diagnosis revealed a significant increase with more advanced stages (*p*_trend_ = 0.006).

**FIGURE 1:**
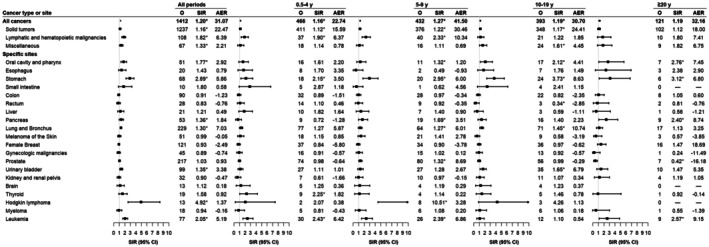
Risk of second primary cancers among 7556 six‐month survivors of PGI‐NHL, overall and by latency time. *Statistically significant standardized incidence ratios are indicated. AER, absolute excess risk; CI, confidence interval; O, observed; PGI‐NHL, primary gastrointestinal non‐Hodgkin lymphoma; SIR, standardized incidence ratio.

PGI‐NHL survivors exhibited significantly elevated risks for both solid tumors (SIR = 1.16, 95% CI = 1.10–1.23) and hematolymphoid malignancies (SIR = 1.82, 95% CI = 1.49–2.19) (Figure 1). Among specific malignancies, over a twofold increased risk compared to the general population was observed for gastric cancer (SIR = 2.89, 95% CI = 2.25–3.67), Hodgkin lymphoma (SIR = 4.92, 95% CI = 2.62–8.42), and leukemia (SIR = 2.05, 95% CI = 1.62–2.56). Additionally, significantly elevated risks were observed for cancers of the oral cavity and pharynx, pancreas, bladder, and lung. Lung cancer and gastric cancer were the primary contributors to the AER, accounting for 23% and 19% of all excess SPC cases, respectively. Latency analysis revealed that the elevated overall risk of SPCs remained stable over time, with SIRs of 1.16, 1.27, 1.19, and 1.19 observed during the 0.5–4, 5–9, 10–19, and ≥ 20 year intervals after PGI‐NHL diagnosis, respectively (*p*_heterogeneity_ = 0.59). Significantly elevated SIRs for gastric cancer, thyroid cancer, and leukemia emerged within the first 0.5–4 years, with the thyroid cancer risk subsequently returning to baseline population levels. In contrast, the risk of developing pancreatic cancer, prostate cancer, Hodgkin lymphoma, oral and pharyngeal cancers, lung cancer, and bladder cancer was elevated only during later follow‐up periods. Notably, 20‐year survivors of PGI‐NHL were at heightened risk for oral and pharyngeal cancers, gastric cancer, pancreatic cancer, and leukemia. Gastric cancer exhibited a consistently elevated risk across all latency periods, including a three‐fold increase beyond 20 years of follow‐up (SIR = 3.12, 95% CI = 1.15–6.80).

**FIGURE 2:**
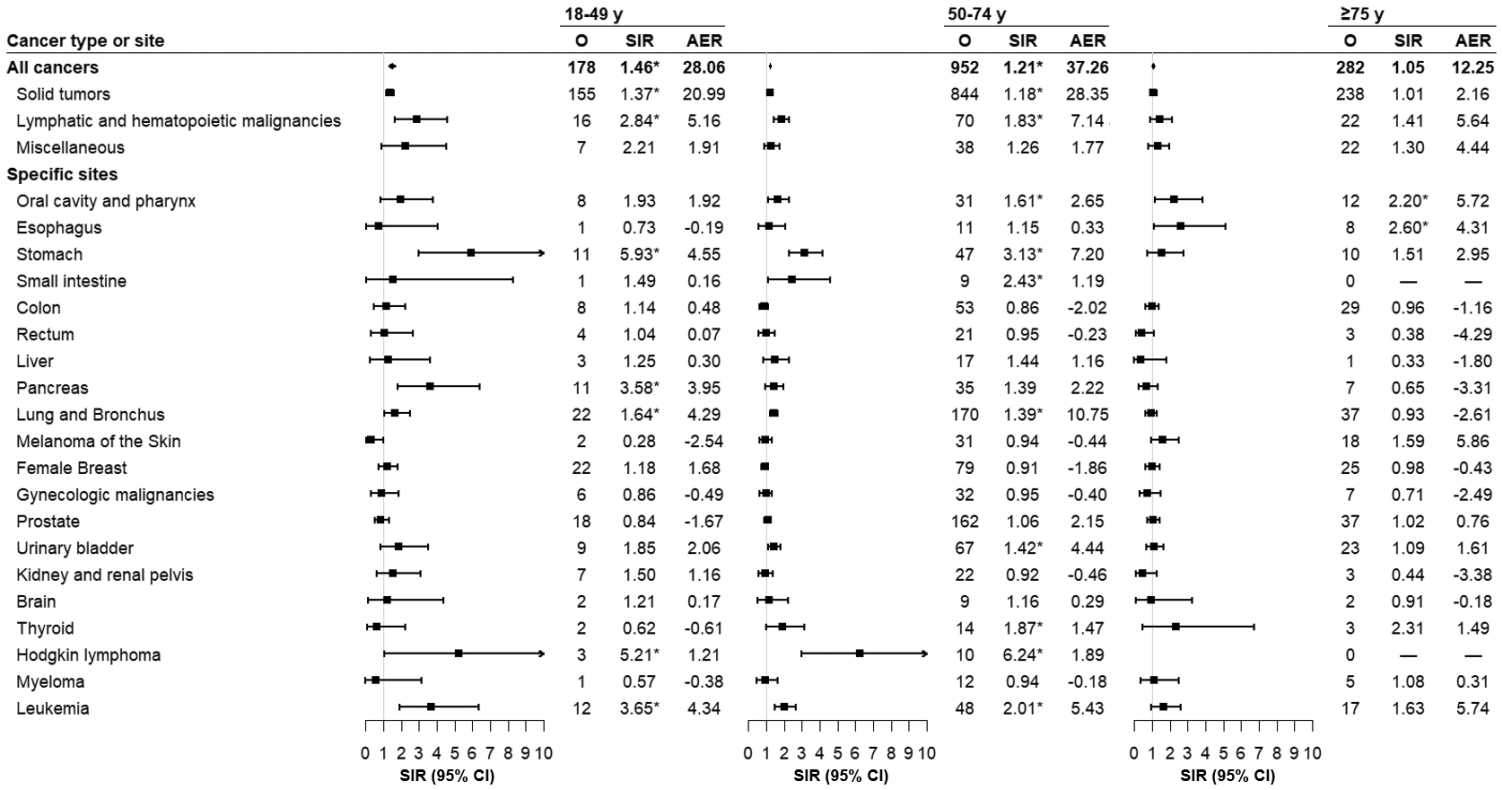
Risk of second primary cancers among 7556 six‐month survivors of PGI‐NHL by age at diagnosis. *Statistically significant standardized incidence ratios are indicated. AER, absolute excess risk; CI, confidence interval; O, observed; PGI‐NHL, primary gastrointestinal non‐Hodgkin lymphoma; SIR, standardized incidence ratio.

The age‐stratified risk profiles of overall and site‐specific SPCs are summarized in Figure 2. Compared with the general population, significantly elevated SPC risks were observed in patients diagnosed with PGI‐NHL at ages 18–49 years (SIR = 1.46, 95% CI = 1.26–1.70) and 50–74 years (SIR = 1.21, 95% CI = 1.14–1.29). Overall, a declining trend in SPC risk with increasing age was observed (*p*_trend_ < 0.001), with the reduction more prominent for hematolymphoid malignancies than for solid tumors. In the site‐specific analysis, the variation in risk across different age groups was heterogeneous. While leukemia risk was significantly increased in patients under 75, excess esophageal cancer risk was confined to those aged 75 and above. Notably, the SIR of pancreatic cancer was more than five times higher in individuals aged 18–49 years (SIR = 3.58, 95% CI = 1.79–6.41) compared to those aged 75 years and older (SIR = 0.65, 95% CI = 0.26–1.34).

**FIGURE 3:**
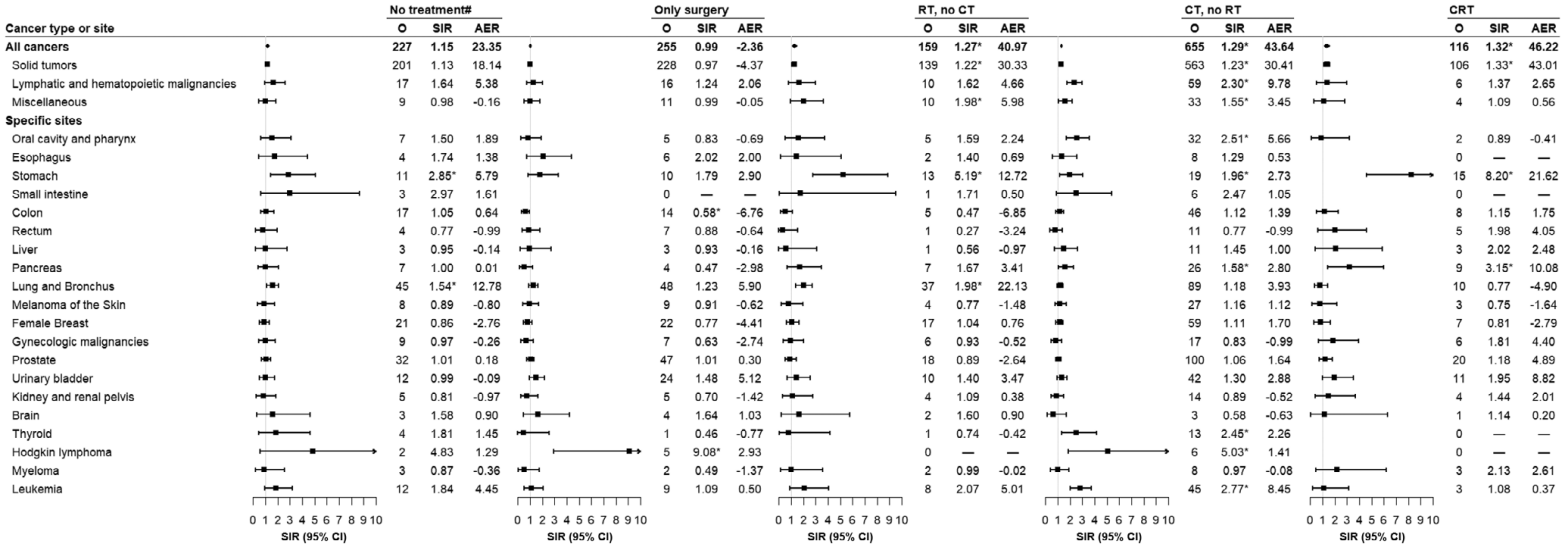
Risk of second primary cancers among 7556 six‐month survivors by treatment modality. *Statistically significant standardized incidence ratios are indicated. ^#^Includes untreated individuals and those with unknown treatment status. AER, absolute excess risk; CI, confidence interval; CRT, chemoradiotherapy; CT, chemotherapy; O, observed; PGI‐NHL, primary gastrointestinal non‐Hodgkin lymphoma; RT, radiotherapy; SIR, standardized incidence ratio.

In our cohort, more than half of the patients received CT, either alone (46%) or in combination with RT (8%). While all treatment modalities (except surgery alone) were associated with elevated risks of SPCs, distinct patterns emerged across the different therapeutic strategies (Figure 3). The most pronounced overall risk elevation was observed in the CRT subgroup (SIR = 1.32, 95% CI = 1.09–1.58), largely driven by an eightfold increase in gastric cancer (SIR = 8.20, 95% CI = 4.59–13.53) and a threefold increase in pancreatic cancer incidence (SIR = 3.15, 95% CI = 1.44–5.98) compared to the general population. Significantly increased risks of cancers of the oral cavity and pharynx, thyroid cancer, and leukemia were observed only in patients who received CT alone, while the risk of pancreatic cancer was elevated in those treated with CT, regardless of RT. In the RT‐only and untreated/treatment status unknown groups, significantly elevated risks of secondary gastric and lung cancers were observed. The SIR for gastric cancer was higher in the RT‐only group than in the untreated/treatment status unknown group (SIR = 5.19 [95% CI = 2.76–8.88] vs. 2.85 [95% CI = 1.42–5.09], *p*_heterogeneity_ = 0.14), and a similar trend was seen for lung cancer (SIR = 1.98 [95% CI = 1.39–2.72] vs. SIR = 1.54 [95% CI = 1.12–2.06], *p*_heterogeneity_ = 0.26), though neither comparison was statistically significant.

**FIGURE 4:**
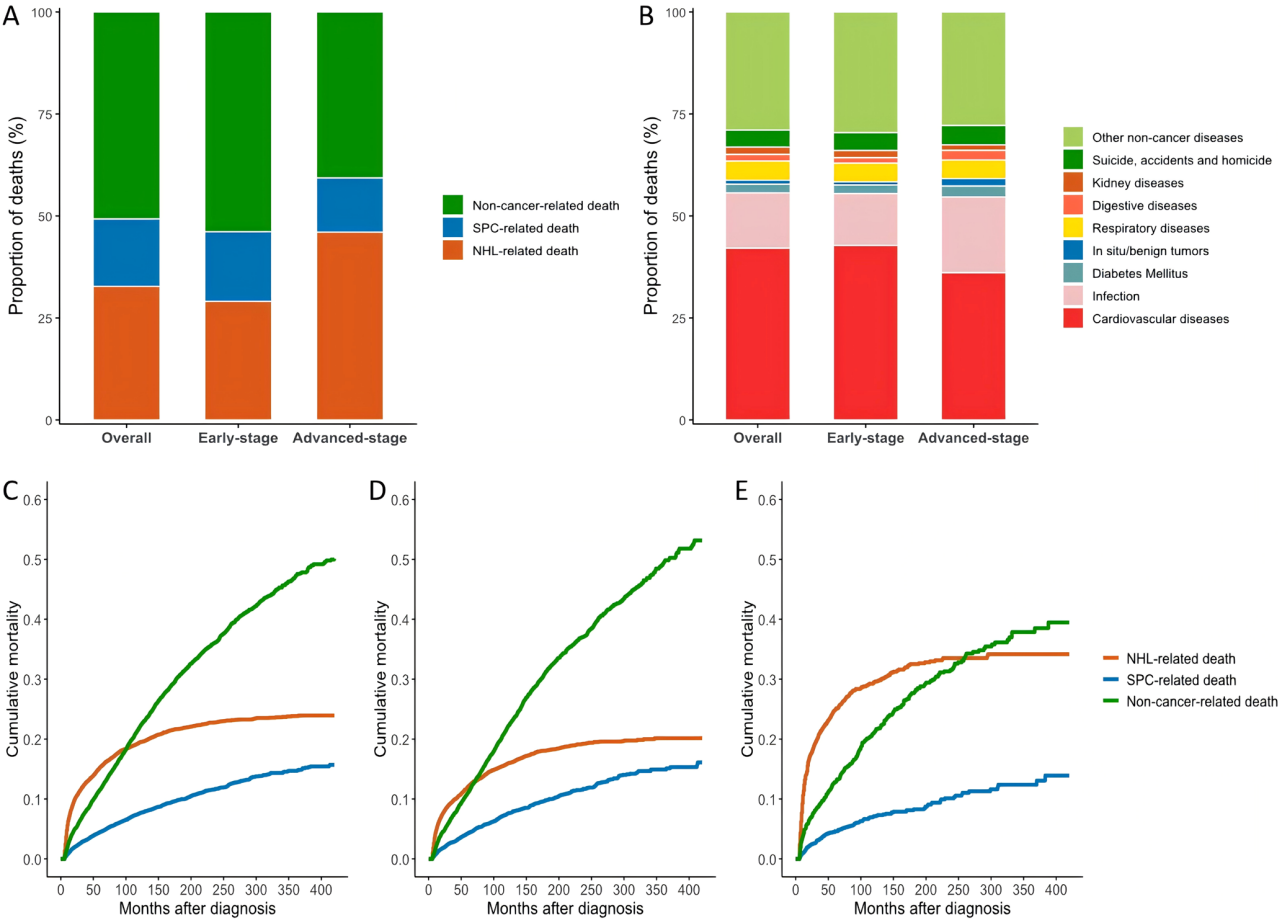
Proportion of deaths and cumulative mortality among 7556 six‐month survivors of PGI‐NHL. (A) Proportional distribution of all‐cause deaths. (B) Proportional distribution of non‐cancer‐related deaths. (C) Cumulative mortality in the overall cohort. (D) Cumulative mortality in early‐stage (stage I–II) PGI‐NHL patients. (E) Cumulative mortality in advanced‐stage (stage III–IV) PGI‐NHL patients. PGI‐NHL, primary gastrointestinal non‐Hodgkin lymphoma.

The cohort experienced 4949 deaths during the study period, with a median survival time of 138 months (95% CI = 134–143). The 5‐, 10‐, and 20‐year cumulative mortality rates were 28.9% (95% CI = 27.9%–29.9%), 45.8% (95% CI = 44.6%–46.9%), and 69.1% (95% CI = 67.8%–70.3%), respectively. In early‐stage (stage I–II) patients, a greater proportion of deaths were attributed to non‐cancer causes (53.9%) than to NHL (29.0%) or secondary malignancies (17.1%). In contrast, in patients with advanced‐stage (stage III–IV) disease, NHL emerged as the leading cause of death (46.0%), followed by non‐cancer mortality (40.7%) (Figure 4A). Cardiovascular diseases were the primary non‐cancer mortality contributor irrespective of cancer stage. Infections accounted for an increasing proportion of non‐cancer deaths as the disease stage progressed, rising from 12.7% in early‐stage patients to 18.6% in those with advanced‐stage disease (Figure 4B). Cumulative mortality analysis revealed that, in the overall cohort (Figure 4C), deaths from non‐cancer causes exceeded NHL‐related deaths within the 5–10 year follow‐up period, with an earlier shift in early‐stage patients (Figure 4D). In advanced‐stage patients, however, NHL remained the leading cause of death for up to 20 years post‐diagnosis (Figure 4E).

**FIGURE 5:**
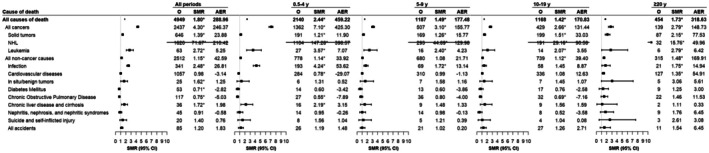
Standardized mortality ratios for specific causes of death among 7556 six‐month survivors of PGI‐NHL, overall and by latency time. *Statistically significant standardized mortality ratios are indicated. AER, absolute excess risk; CI, confidence interval; O, observed; PGI‐NHL, primary gastrointestinal non‐Hodgkin lymphoma; SMR, standardized mortality ratio.

PGI‐NHL patients exhibited significantly elevated all‐cause mortality compared to the general population (SMR = 1.80, 95% CI = 1.75–1.85), with the excess sustained over the entire follow‐up period (Figure 5). This increased mortality was attributable not only to all cancers combined (SMR = 4.30, 95% CI = 4.13–4.47) but also to non‐cancer causes (SMR = 1.15, 95% CI = 1.10–1.19), especially among younger patients (18–49 years: SMR = 3.27, 95% CI = 2.92–3.64; 50–74 years: SMR = 1.14, 95% CI = 1.07–1.20) (Table S1). Overall, significant excess mortality was observed for NHL, leukemia, solid tumors, infections, in situ/benign tumors, and chronic liver disease/cirrhosis, whereas reduced mortality was noted for diabetes mellitus and chronic obstructive pulmonary disease. Latency analysis revealed persistent excess mortality from NHL, leukemia, solid tumors, and infections extending beyond 20 years post‐diagnosis. NHL‐related mortality declined significantly over time (*p*_trend_ < 0.001), in contrast to the marked increase in mortality from solid tumors (*p*_trend_ < 0.001). Additionally, cardiovascular mortality was 22% lower than expected during the first 5 years after diagnosis (SMR = 0.78, 95% CI = 0.69–0.88), but increased by 35% after 20 years (SMR = 1.35, 95% CI = 1.13–1.61). Notably, patients aged 18–49 years had a 62% higher risk of cardiovascular mortality than the general population (SMR = 1.62, 95% CI = 1.24–2.08).

## 4 Discussion

This is the first comprehensive assessment of SPC incidence and survival outcomes among PGI‐NHL survivors, using population‐based data with over 40 years of follow‐up. Our study identified a 1.20‐fold increased risk of SPCs and a 1.80‐fold higher all‐cause mortality compared to the general US population. While SPC patterns have been well characterized in general NHL cohorts, data on this clinically distinct extranodal subgroup remain limited. Few large‐scale studies with prolonged follow‐up have been conducted, and most available evidence focuses narrowly on specific anatomical sites or histologic subtypes. Tajika et al. [16] first reported a significantly elevated SPC risk (SIR = 3.99) in 146 patients with gastric MALT lymphoma. Subsequent studies by Amiot et al. [18] and Yang et al. [17] observed more modest increases, with SIRs of 1.71 and 1.46, respectively. To date, the SEER analysis by Yin et al. [19] remains the only study quantifying gastrointestinal DLBCL SPC risk, reporting an SIR of 1.16. This current analysis, including over 1000 ≥ 20‐year survivors of PGI‐NHL, expands the characterization of future cancer risk profiles and delineates variations across patient demographics, treatment exposures, and latency periods.

Gastric cancer demonstrated a consistently elevated risk across all follow‐up periods, with a three‐fold increase beyond 20 years post‐diagnosis. The excess risk was more pronounced in a French cancer registry study, where 175 gastric MALT lymphoma patients showed a 16‐fold higher risk of secondary gastric cancer, despite a relatively short follow‐up period (median 3.5 years) [18]. In contrast, a cohort of 18,812 nodal NHL survivors showed no significant increase in secondary gastric cancer risk [21]. This disparity suggests the distinct pathogenesis of secondary malignancies in PGI‐NHL survivors. Shared environmental factors and pathogenic mechanisms likely contribute to the elevated risk of gastric cancer and other malignancies. Both gastric adenocarcinoma and MALT lymphoma are strongly associated with *Helicobacter pylori* infection, with documented cases of their synchronous occurrence in chronic inflammatory environments [22, 23]. Additionally, Epstein–Barr virus (EBV), an oncogenic factor in Burkitt lymphoma, is implicated in EBV‐positive gastric and oropharyngeal carcinomas, as well as Hodgkin lymphoma [24]. These environmental triggers may interact with genetic susceptibilities. Frequent mutations in tumor suppressor genes (e.g., p53 and p16), defects in DNA repair pathways, and chromosomal abnormalities such as trisomy 3p collectively establish a tumor‐prone phenotype, predisposing survivors to both lymphoid malignancies and a spectrum of solid tumors [16].

The immune dysregulation inherent to PGI‐NHL, compounded by treatment‐induced immunosuppression, may contribute to the elevated risk of SPCs. The increased risk of thyroid cancer was limited to the first 5 years after PGI‐NHL diagnosis, a phenomenon not previously reported. Though chance cannot be entirely ruled out, the 6.9‐fold higher incidence of thyroid cancer observed in kidney transplant recipients undergoing immunosuppression, particularly with a clustering of early‐onset cases, suggests that immunosuppression may expedite the progression of latent disease to a clinically detectable stage [25]. The enhanced immune suppression induced by alkylating agents may further account for the markedly elevated risk of secondary thyroid cancer observed in patients receiving CT. Our analysis corroborates prior population‐based findings by Yang et al. [17] and Yin et al. [19], confirming heightened lung cancer susceptibility in PGI‐NHL survivors. Immunological alterations associated with lymphomas likely contribute, as lung cancer occurs disproportionately in immunosuppressed patients, with inflammation and infections further increasing the risk [26, 27]. PGI‐NHL survivors exhibited an elevated risk of HL. While shared viral cofactors and immune dysregulation may explain the sequential development of distinct lymphoid neoplasms, diagnostic misclassification remains a concern due to the overlapping morphologies between recurrent NHL and secondary HL, as well as the under‐recognition of histologic transformation [21].

Current therapeutic approaches for PGI‐NHL include watchful waiting, *Helicobacter pylori* eradication, CT, RT, immunotherapy (e.g., rituximab), surgical resection, and combined‐modality treatments [2]. Carcinogenesis at specific tumor sites is a known late effect of CT, due to DNA damage and mutagenic lesions that promote cellular transformation [28]. Our analysis identified CT as the sole modality significantly associated with an elevated leukemia risk, consistent with prior reports implicating anthracyclines and alkylating agents in therapy‐related myeloid neoplasms [21, 29]. Leukemogenic risk was significantly elevated within the first decade following PGI‐NHL diagnosis before declining, but a statistically significant resurgence beyond 20 years underscores the need for lifelong surveillance. An elevated risk of pancreatic cancer emerged beyond 5 years after PGI‐NHL diagnosis and was significant only among patients treated with CT. Although this association has not been previously reported and lacks a confirmed treatment link, evidence from an international case–control study in a Hodgkin's lymphoma cohort demonstrated a dose‐dependent increase in pancreatic cancer risk with alkylating agent–based regimens [30]. Given the therapeutic parallels between NHL and HL, similar treatment‐related risks may exist.

The carcinogenic potential of RT remains incompletely characterized, particularly among PGI‐NHL survivors. In our analysis, SIRs for gastric and lung cancers were higher in RT‐only groups compared to untreated cohorts, although the differences did not reach statistical significance, likely due to limited power from the small sample size. Gamma radiation has been implicated in increased stomach cancer risk [31]. In population‐based studies of Hodgkin lymphoma and testicular cancer, subdiaphragmatic RT has been significantly associated with elevated risks of secondary gastrointestinal malignancies [32, 33]. While prior investigations in NHL have suggested a link between RT and lung carcinogenesis, the contribution of RT in our cohort should be interpreted with caution, given the limited doses and constrained fields characteristic of modern PGI‐NHL protocols. Moreover, epidemiological evidence indicates that combining RT or CT with smoking further amplifies lung cancer risk [34], but smoking data were unavailable in our study. Especially high risks for secondary gastric and pancreatic cancers were observed among patients receiving CRT. The joint effect of these two treatments was found to be greater than multiplicative for development of secondary gastric and pancreatic cancers in HL survivors [30, 33]. Further investigation, incorporating detailed exposure parameters, is warranted to delineate the underlying synergistic effects of CT and RT in the development of SPCs among PGI‐NHL survivors.

Our findings show a significant increase in SPC risk with more advanced PGI‐NHL stages, despite a higher competing risk of death. This elevated risk likely reflects the intensified therapeutic exposure required for advanced disease. The risk of developing SPCs was inversely associated with patient age at PGI‐NHL diagnosis. Previous studies have shown that, compared to adults and the elderly, adolescents with extranodal DLBCL originating from the gastrointestinal tract and skeletal tissues are at a higher risk for developing SPCs [19]. Cancer treatment in younger individuals is often more aggressive, which may increase carcinogenic risk. Additionally, longer survival times in younger cancer survivors allow for the emergence of neoplasms with long latencies. An exception to this trend was noted in esophageal cancer, where a significantly higher risk was observed exclusively in those aged ≥ 75 years. The mechanisms of carcinogenesis may differ by site, influenced by both etiological factors and treatment‐related effects. These findings support a tailored approach to survivorship care, emphasizing risk‐adapted SPC screening and proactive management of treatment sequelae.

The occurrence of additional neoplasms in PGI‐NHL patients has been a persisting clinical concern. Historically, studies prior to 2000 reported no excess cancer risk in gastric MALT lymphoma patients versus general populations [35, 36, 37]. However, a contemporary population‐based study of patients diagnosed with gastric MALTs between 2000 and 2014 revealed a 46% increase in the incidence of SPCs [17]. Our period analysis supports this evolving risk profile, demonstrating a significant rise in overall SPC risk in the post‐2001 era, with particularly notable increases in secondary thyroid cancer and leukemia. The mechanisms underlying this evolving risk profile are likely multifactorial, potentially involving prolonged patient survival due to therapeutic advancements, enhanced diagnostic sensitivity, and the specific late effects of modern therapies. Following rituximab's integration into first‐line immunochemotherapy regimens for CD20+ B‐cell lymphoma in the early 2000s, concerns have arisen regarding its potential association with elevated SPC risk. Prolonged B‐cell depletion and T‐cell inactivation induced by rituximab may impair immune surveillance and sustain an immunosuppressive state [38]. While definitive evidence linking rituximab to increased cancer susceptibility remains limited, several studies have reported elevated risks of secondary thyroid cancer, melanoma, and acute myeloid leukemia among NHL survivors in the post‐rituximab era [38, 39, 40, 41]. Looking ahead, the clinical integration of targeted agents and novel immunotherapies—such as immune checkpoint inhibitors and CAR‐T cells—has transformed NHL treatment paradigms [42, 43]. These therapies have distinct mechanisms and toxicity profiles that may influence long‐term SPC risks compared to traditional modalities. Consequently, ongoing surveillance through extended follow‐up studies and expanded registries will be essential to characterize the evolving spectrum of survivorship risks in this new therapeutic era.

To our knowledge, this represents the first large‐scale population‐based study delineating cause‐specific mortality patterns among PGI‐NHL survivors. The most common causes of death other than NHL were SPCs, cardiovascular diseases (CVD), and infections. Among patients with early‐stage disease, non‐cancer causes accounted for the majority of deaths. Competing risk regression showed that the cumulative incidence of non‐cancer deaths surpassed that of NHL within 5–10 years post‐diagnosis. CVD is the leading cause of non‐cancer mortality and one of the most concerning late effects of cancer therapies. Cumulative, dose‐dependent cardiotoxic effects—including anthracycline‐related myocardial dysfunction and radiotherapy‐induced vascular injury—contribute to the elevated cardiovascular mortality risk in NHL survivors [44, 45, 46]. Given that CVD progresses gradually, it is typically detected after more than 15 years of follow‐up [44, 47]. In our study, cardiovascular mortality in the first 5 years post‐diagnosis was 22% lower than in the general population, possibly due to enhanced cardiac monitoring and competing mortality from aggressive PGI‐NHL progression. However, this initial protective pattern reversed with prolonged follow‐up, as survivors demonstrated a 35% excess cardiovascular mortality risk at ≥ 20 years post‐diagnosis. This shift highlights the need for heightened clinical attention to delayed cardiovascular sequelae in PGI‐NHL management.

While previous studies have focused on short‐term infection risks [48, 49], our longitudinal cohort revealed persistently elevated infectious mortality rates lasting more than two decades post‐diagnosis. The burden of infection‐related mortality was more evident among advanced‐stage PGI‐NHL patients, likely due to intensified treatment regimens and progressive immune dysfunction that increase susceptibility to opportunistic infections. Emerging evidence supports an association between hepatitis C virus infection and specific B‐cell NHL subtypes, particularly DLBCL, marginal zone lymphoma, and lymphoplasmacytic lymphoma [50]. The observed excess mortality from chronic liver disease and cirrhosis may be partially attributable to hepatitis virus infection and hepatotoxic drug exposures [51, 52], highlighting the need for hepatitis screening and regular liver function tests as per physician recommendations. Long‐term follow‐up also revealed sustained excess mortality from secondary solid tumors and leukemia among PGI‐NHL patients, with the SMR for solid tumors rising over time. Clinicians should adopt lifelong follow‐up protocols integrating multidisciplinary strategies and early intervention to ensure optimal care for PGI‐NHL survivors.

Several limitations inherent in registry‐based studies should be considered when interpreting our findings. First, the SEER database lacks key confounders such as family history, viral infections, and lifestyle factors (e.g., alcohol and tobacco use), which preclude clear delineation of their independent roles in SPC development and mortality. Second, treatment data are incomplete regarding specifics of regimens (drug classes, doses) and radiation fields, constraining interpretation of treatment‐related risks and potentially biasing association estimates. Third, potential underreporting of secondary malignancies or mortality due to patient emigration from registry catchment areas may lead to underestimated SIR/SMR values. Nonetheless, this large‐scale cohort study with extended follow‐up addresses a significant gap in the epidemiology of secondary cancers among long‐term PGI‐NHL survivors and provides the first comprehensive cause‐specific mortality profile for this population. Future multicenter studies incorporating thorough individual data are essential to further explore the underlying explanations for the observed outcomes.

## 5 Conclusions

This population‐based cohort study underscores the persistent excess risks of SPCs and cause‐specific mortality among PGI‐NHL survivors, extending decades beyond initial diagnosis. Younger patients and those treated with chemoradiotherapy warrant intensified surveillance for SPCs. A long‐term multidisciplinary survivorship strategy, incorporating risk‐adapted cancer management and proactive control of non‐cancer complications, is essential for optimizing health outcomes.

## Author Contributions

**Jiahe Wu:** conceptualization (equal), data curation (equal), formal analysis (equal), methodology (equal), visualization (equal), writing – original draft (equal). **Jiangping Yang:** data curation (equal), formal analysis (equal), writing – original draft (equal). **Yuxi Ding:** data curation (equal), formal analysis (equal), validation (equal). **Jili Wang:** validation (equal), visualization (equal). **Xiaofei Cheng:** validation (equal), visualization (equal). **Liangshun You:** conceptualization (equal), project administration (equal), writing – review and editing (equal). **Feng Zhao:** conceptualization (equal), funding acquisition (equal), project administration (equal), supervision (equal), writing – review and editing (equal).

## Ethics Statement

SEER data is deidentified public databases. No institutional review board approval was required for this analysis.

## Conflicts of Interest

The authors declare no conflicts of interest.

## Supporting information


**Figure S1:** Risk of second primary cancers among 7556 six‐month survivors of PGI‐NHL by treatment era (pre‐2001 vs. post‐2001).

**Figure S2:** Risk of second primary cancers among 7556 six‐month survivors of PGI‐NHL by histologic subtype.

**Table S1:** Standardized mortality ratios for 7556 six‐month survivors of PGI‐NHL according to baseline characteristics.

## Data Availability

The data of this article are available at: https://seer.cancer.gov/.
