# Supplementary figures and images for: Second Cancer Incidence and Cause‐Specific Mortality in Primary Gastrointestinal Non‐Hodgkin Lymphoma Survivors: A Population‐Based Cohort Study
Source: Cancer Med. 2025 Nov 20;14(22):e71405. doi: 10.1002/cam4.71405 (PMC12631535; doi:10.1002/cam4.71405)

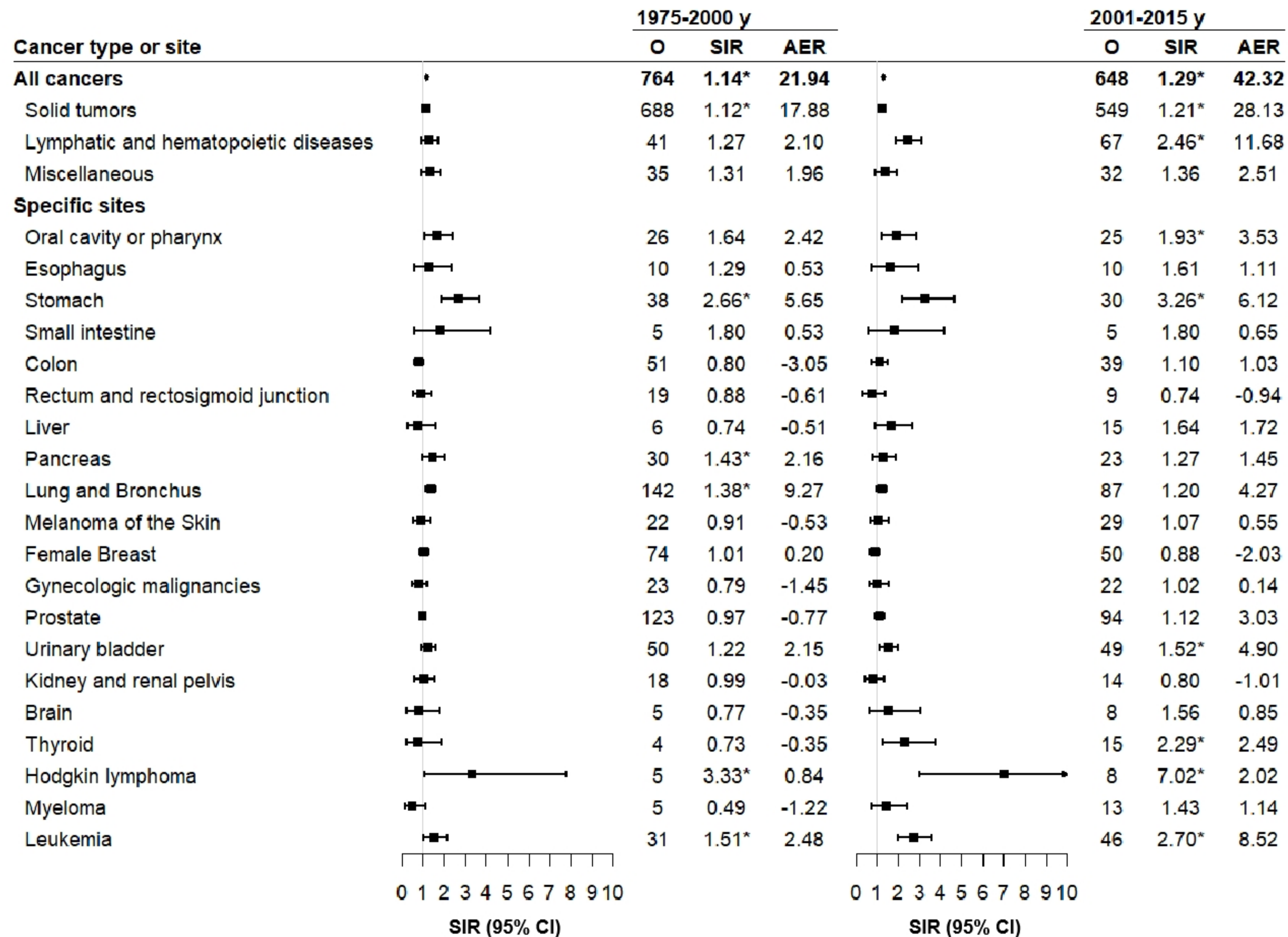

Supplement: Supplementary file 1 — Figure S1: Risk of second primary cancers among 7556 six‐month survivors of PGI‐NHL by treatment era (pre‐2001 vs. post‐2001). [file CAM4-14-e71405-s002.pdf]

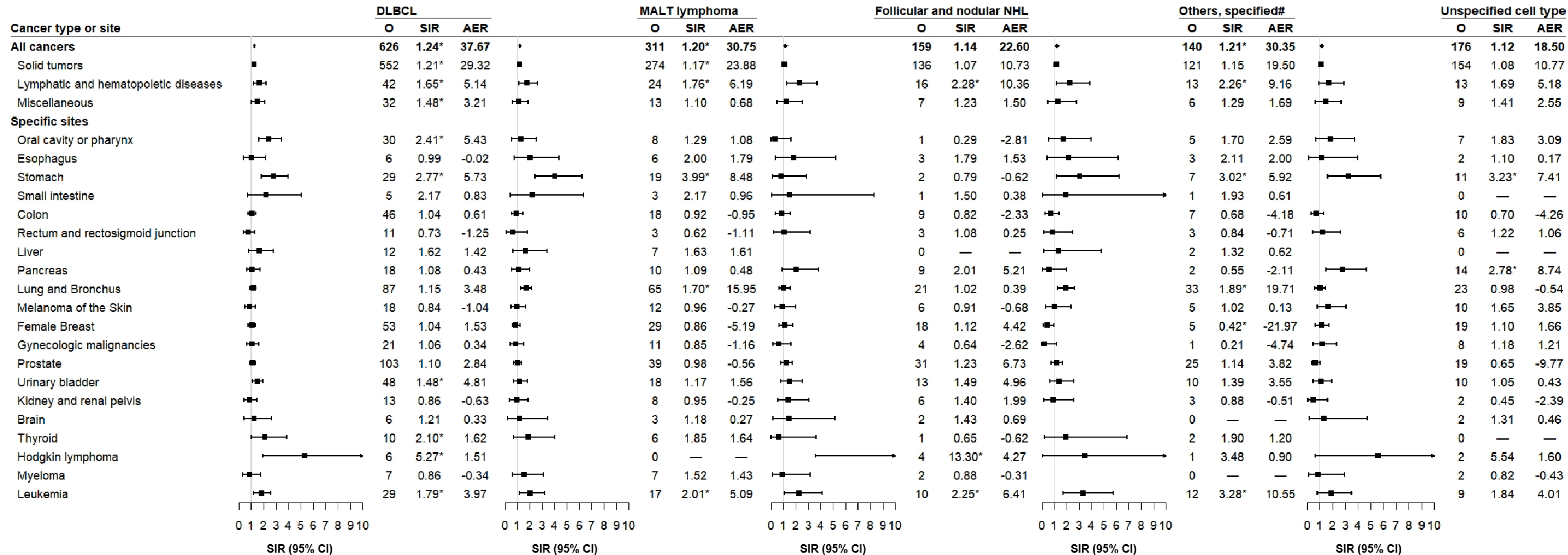

Supplement: Supplementary file 2 — Figure S2: Risk of second primary cancers among 7556 six‐month survivors of PGI‐NHL by histologic subtype. [file CAM4-14-e71405-s001.pdf]
