# Supplementary material for: Second Cancer Incidence and Cause‐Specific Mortality in Primary Gastrointestinal Non‐Hodgkin Lymphoma Survivors: A Population‐Based Cohort Study
Source: Cancer Med. 2025 Nov 20;14(22):e71405. doi: 10.1002/cam4.71405 (PMC12631535; doi:10.1002/cam4.71405)
Supplement: Supplementary file 3 — Table S1: Standardized mortality ratios for 7556 six‐month survivors of PGI‐NHL according to baseline characteristics. [file CAM4-14-e71405-s003.docx]

**Supplementary table 1. Standardized mortality ratios for 7556 six-month survivors of PGI-NHL according to baseline characteristics**

|  | **NHL** | |  | **Second primary cancers** | |  |  | **All non-cancer causes** |  | **Cardiovascular diseases** | |  | **Infection** | |  | | | **Chronic liver disease and cirrhosis** | |  |
| --- | --- | --- | --- | --- | --- | --- | --- | --- | --- | --- | --- | --- | --- | --- | --- | --- | --- | --- | --- | --- |
| **Patient characteristics** | **O** | **SMR(95% CI)** |  | **O** | **SMR(95% CI)** |  | **O** | **SMR(95% CI)** |  | **O** | **SMR(95% CI)** |  | **O** | **SMR(95% CI)** | |  | **O** | | **SMR(95% CI)** | |
| **Sex** |  |  |  |  |  |  |  |  |  |  |  |  |  |  | |  |  | |  | |
| Males | 905 | **67.35 (63.03, 71.88)** |  | 494 | **1.50 (1.37, 1.64)** |  | 1396 | **1.21 (1.15, 1.27)** |  | 537 | 0.94 (0.86, 1.02) |  | 251 | **3.38 (2.98, 3.83)** | |  | 16 | | 1.13 (0.65, 1.83) | |
| Females | 715 | **78.01 (72.39, 83.94)** |  | 323 | **1.50 (1.34, 1.67)** |  | 1116 | **1.08 (1.02, 1.15)** |  | 520 | 1.03 (0.94, 1.12) |  | 90 | **1.42 (1.14, 1.75)** | |  | 20 | | **2.95 (1.80, 4.56)** | |
| **Year of PGI-NHL diagnosis** |  |  |  |  |  |  |  |  |  |  |  |  |  |  | |  |  | |  | |
| 1975-1984 | 284 | **96.54 (85.64, 108.45)** |  | 144 | **1.91 (1.61, 2.25)** |  | 379 | **1.27 (1.14, 1.40)** |  | 183 | 1.00 (0.86, 1.15) |  | 41 | **2.01 (1.44, 2.73)** | |  | 6 | | 2.07 (0.76, 4.49) | |
| 1985-1994 | 465 | **76.95 (70.12, 84.27)** |  | 242 | **1.72 (1.51, 1.95)** |  | 728 | **1.33 (1.23, 1.43)** |  | 306 | 1.03 (0.92, 1.16) |  | 135 | **3.52 (2.96, 4.17)** | |  | 7 | | 1.44 (0.58, 2.97) | |
| 1995-2004 | 478 | **61.93 (56.51, 67.74)** |  | 255 | **1.40 (1.23, 1.58)** |  | 827 | **1.12 (1.04, 1.20)** |  | 333 | 0.96 (0.86, 1.07) |  | 118 | **2.60 (2.15, 3.11)** | |  | 13 | | **1.97 (1.05, 3.37)** | |
| 2005-2015 | 393 | **66.60 (60.18, 73.52)** |  | 176 | **1.21 (1.04, 1.41)** |  | 578 | 0.96 (0.89, 1.04) |  | 235 | 0.93 (0.81, 1.05) |  | 47 | **1.41 (1.04, 1.88)** | |  | 10 | | 1.52 (0.73, 2.79) | |
| **Age at PGI-NHL diagnosis,y** |  |  |  |  |  |  |  |  |  |  |  |  |  |  | |  |  | |  | |
| 18-49 | 162 | **130.88 (111.50, 152.66)** |  | 83 | **2.25 (1.79, 2.78)** |  | 320 | **3.27 (2.92, 3.64)** |  | 61 | **1.62 (1.24, 2.08)** |  | 160 | **23.98 (20.41, 28.00)** | |  | 8 | | 1.93 (0.84, 3.81) | |
| 50-74 | 901 | **66.70 (62.42, 71.20)** |  | 508 | **1.51 (1.38, 1.64)** |  | 1224 | **1.14 (1.07, 1.20)** |  | 497 | 0.95 (0.87, 1.04) |  | 118 | **1.84 (1.52, 2.20)** | |  | 24 | | **1.76 (1.13, 2.62)** | |
| ≥75 | 557 | **70.89 (65.12, 77.03)** |  | 226 | **1.33 (1.16, 1.52)** |  | 968 | 0.96 (0.90, 1.02) |  | 499 | 0.96 (0.88, 1.05) |  | 63 | 0.95 (0.73, 1.21) | |  | 4 | | 1.25 (0.34, 3.20) | |
| **Race** |  |  |  |  |  |  |  |  |  |  |  |  |  |  | |  |  | |  | |
| White | 1335 | **66.47 (62.95, 70.13)** |  | 690 | **1.47 (1.36, 1.59)** |  | 2077 | **1.09 (1.05, 1.14)** |  | 880 | **0.94 (0.87, 1.00)** |  | 269 | **2.34 (2.07, 2.63)** | |  | 32 | | **1.78 (1.22, 2.51)** | |
| Black | 76 | **128.20(101.01,160.46)** |  | 45 | **1.57 (1.15, 2.10)** |  | 142 | **1.38 (1.17, 1.63)** |  | 53 | 1.06 (0.79, 1.38) |  | 39 | **5.15 (3.66, 7.04)** | |  | 2 | | 2.01 (0.24, 7.26) | |
| Other/unknown | 209 | **108.51 (94.29, 124.26)** |  | 82 | **1.76 (1.40, 2.18)** |  | 293 | **1.60 (1.42, 1.79)** |  | 124 | **1.39 (1.16, 1.66)** |  | 33 | **2.23 (1.54, 3.14)** | |  | 2 | | 1.01 (0.12, 3.64) | |
| **Stage** |  |  |  |  |  |  |  |  |  |  |  |  |  |  | |  |  | |  | |
| I | 547 | **45.89 (42.13, 49.90)** |  | 381 | **1.36 (1.22, 1.50)** |  | 1274 | **1.10 (1.04, 1.16)** |  | 544 | 0.98 (0.90, 1.07) |  | 164 | **2.29 (1.95, 2.67)** | |  | 13 | | 1.24 (0.66, 2.12) | |
| II | 374 | **84.44 (76.10, 93.44)** |  | 162 | **1.51 (1.29, 1.76)** |  | 435 | 1.04 (0.95, 1.14) |  | 187 | 0.92 (0.80, 1.07) |  | 53 | **2.02 (1.51, 2.64)** | |  | 6 | | 1.41 (0.52, 3.07) | |
| III | 75 | **117.95(92.77, 147.85)** |  | 27 | **1.74 (1.15, 2.53)** |  | 76 | 1.21 (0.95, 1.52) |  | 28 | 0.94 (0.63, 1.36) |  | 13 | **3.31 (1.76, 5.66)** | |  | 1 | | 1.63 (0.04, 9.06) | |
| IV | 351 | **148.20 (133.10, 164.54)** |  | 96 | **1.66 (1.34, 2.02)** |  | 301 | **1.40 (1.25, 1.57)** |  | 108 | 1.05 (0.87, 1.27) |  | 57 | **4.30 (3.26, 5.57)** | |  | 8 | | **3.21 (1.39, 6.32)** | |
| Unknown | 273 | **84.00 (74.33, 94.57)** |  | 151 | **1.83 (1.55, 2.15)** |  | 426 | **1.27 (1.15, 1.40)** |  | 190 | 0.98 (0.85, 1.13) |  | 54 | **2.42 (1.82, 3.16)** | |  | 8 | | **2.58 (1.12, 5.09)** | |
| **PGI-NHL histopathology** |  |  |  |  |  |  |  |  |  |  |  |  |  |  | |  |  | |  | |
| DLBCL | 852 | **85.21 (79.58, 91.13)** |  | 373 | **1.57 (1.42, 1.74)** |  | 1200 | **1.25 (1.18 ,1.32)** |  | 503 | 1.04 (0.95, 1.14) |  | 196 | **3.20 (2.77, 3.68)** | |  | 18 | | **2.01 (1.19, 3.18)** | |
| MALT lymphoma | 146 | **30.75 (25.96, 36.16)** |  | 144 | **1.24 (1.04, 1.46)** |  | 492 | 1.02 (0.93, 1.12) |  | 199 | 0.93 (0.80, 1.06) |  | 31 | 1.10 (0.75, 1.56) | |  | 8 | | 1.78 (0.77, 3.50) | |
| Follicular and nodular NHL | 127 | **49.99 (41.67, 59.47)** |  | 72 | 1.15 (0.90, 1.45) |  | 173 | **0.73 (0.63, 0.85)** |  | 79 | 0.69 (0.55, 0.86) |  | 16 | 1.11 (0.64, 1.81) | |  | 4 | | 1.60 (0.43, 4.08) | |
| Others, specified | 263 | **117.66 (103.87, 132.77)** |  | 92 | **1.71 (1.38, 2.10)** |  | 251 | **1.20 (1.05, 1.36)** |  | 110 | 1.01 (0.83, 1.22) |  | 35 | **2.60 (1.81, 3.61)** | |  | 3 | | 1.38 (0.28, 4.02) | |
| Unspecified cell type | 232 | **75.33 (65.95, 85.67)** |  | 136 | **1.83 (1.54, 2.17)** |  | 396 | **1.32 (1.19, 1.45)** |  | 166 | 1.05 (0.89, 1.22) |  | 63 | **3.13 (2.40, 4.00)** | |  | 3 | | 1.07 (0.22, 3.12) | |
| **Site of PGI-NHL** |  |  |  |  |  |  |  |  |  |  |  |  |  |  | |  |  | |  | |
| Stomach | 816 | **62.13 (57.94, 66.55)** |  | 503 | **1.60 (1.46, 1.75)** |  | 1561 | **1.21 (1.15, 1.27)** |  | 693 | 1.06 (0.99, 1.15) |  | 182 | **2.21 (1.90, 2.56)** | |  | 22 | | **1.95 (1.22, 2.95)** | |
| Small intestine | 502 | **90.00 (82.30, 98.23)** |  | 181 | **1.33 (1.14, 1.54)** |  | 528 | 1.02 (0.94, 1.11) |  | 217 | **0.86 (0.75, 0.99)** |  | 75 | **2.36 (1.85, 2.96)** | |  | 7 | | 1.23 (0.49, 2.53) | |
| Colon | 250 | **77.90 (68.54, 88.17)** |  | 104 | **1.36 (1.11, 1.65)** |  | 322 | 1.05 (0.94, 1.17) |  | 119 | **0.82 (0.68, 0.99)** |  | 48 | **2.54 (1.87, 3.36)** | |  | 6 | | 1.83 (0.67, 3.99) | |
| Rectum | 52 | **76.11 (56.84, 99.81)** |  | 29 | **1.68 (1.12, 2.41)** |  | 101 | **1.43 (1.16, 1.73)** |  | 28 | 0.83 (0.55, 1.20) |  | 36 | **8.24 (5.77, 11.41)** | |  | 1 | | 1.44 (0.04, 8.02) | |

Abbreviations: DLBCL, diffuse large B-cell lymphoma; MALT, mucosa-associated lymphoid tissue; PGI-NHL, primary gastrointestinal non-Hodgkin lymphoma; SMR, standardized mortality ratio.
